# Baseline Volumetric T2 Relaxation Time Histogram Analysis: Can It Be Used to Predict the Response to Intravenous Methylprednisolone Therapy in Patients With Thyroid-Associated Ophthalmopathy?

**DOI:** 10.3389/fendo.2021.614536

**Published:** 2021-02-25

**Authors:** Ping Liu, Ban Luo, Lang Chen, Qiu-Xia Wang, Gang Yuan, Gui-hua Jiang, Jing Zhang

**Affiliations:** ^1^ Department of Radiology, The Affiliated Tongji Hospital, Tongji Medical College, Huazhong University of Science & Technology, Wuhan, China; ^2^ Department of Medical Imaging, Guangdong Second Provincial General Hospital, Guangzhou, China; ^3^ Department of Ophthalmology, The Affiliated Tongji Hospital, Tongji Medical College, Huazhong University of Science & Technology, Wuhan, China; ^4^ Department of Endocrinology and Metabolism, The Affiliated Tongji Hospital, Tongji Medical College, Huazhong University of Science & Technology, Wuhan, China

**Keywords:** thyroid associated ophthalmopathy, extraocular muscle, T2 relaxation time, intravenous methylprednisolone pulse, treatment response

## Abstract

**Objective:**

Prediction of therapy response to intravenous methylprednisolone pulses (ivMP) is crucial for thyroid-associated ophthalmopathy (TAO). Image histograms may offer sensitive imaging biomarkers for therapy effect prediction. This study aimed to investigate whether pretherapeutic, multiparametric T2 relaxation time(T2RT) histogram features of extraocular muscles (EOMs) can be used to predict therapy response.

**Materials and Methods:**

Forty-five active and moderate-severe TAO patients, who were treated with standard ivMP and underwent orbital MRI before therapy, were retrospectively included in this study. The patients were divided into responsive (n = 24, 48 eyes) and unresponsive group(n = 21, 42 eyes) according to clinical evaluation. Baseline clinical features of patients and histogram-derived T2RT parameters of the EOMs were analyzed and compared. Logistic regression model was conducted to determine independent predictors, and a histogram features nomogram was formulated for personalized prediction.

**Results:**

Responsive group displayed lower values for 5^th^, 10^th^ percentiles (P < 0.050, respectively), and higher values for 75^th^, 90^th^, and 95^th^ percentiles, skewness, entropy, and inhomogeneity (P < 0.050, respectively) than unresponsive group. Multivariate logistic regression analysis showed that 95^th^ percentile of >88.1 [odds ratio (OR) = 12.078; 95% confidence interval (CI) = 3.98–36.655, p < 0.001], skewness of >0.31 (OR = 3.935; 95% CI = 2.28–6.788, p < 0.001) and entropy of >3.41 (OR = 4.375; 95% CI = 2.604–7.351, p < 0.001) were independent predictors for favorable response. The nomogram integration of three independent predictors demonstrated optimal predictive efficiency, with a C-index of 0.792.

**Conclusions:**

Pre-treatment volumetric T2RT histogram features of EOMs could function to predict the response to ivMP in patients with TAO. The nomogram based on histogram features facilitates the selection of patients who will derive maximal benefit from ivMP.

## Introduction

Thyroid-associated ophthalmopathy (TAO) is an autoimmune disorder affecting the orbital and periorbital tissues. Intravenous methylprednisolone pulse (ivMP) remains the first-line treatment for active, moderate-to-severe TAO (1). However, the overall response to ivMP therapy is not very satisfactory (1, 2), many patients exhibit non-response or progression (3). This may relate to other complex pathogenesis of TAO, including helper T-cells, T-regulatory cells, and interleukin-17/23. Thus, targeted biological regimens such as rituximab(anti-CD20) and infliximab (anti-TNF-alpha monoclonal antibodies) may achieve better response than the broad-spectrum methylprednisolone in some cases (4–6). Hence, therapeutic response prediction is essential for patient-tailored treatment, as it helps to identify those patients who will truly benefit from the potentially toxic ivMP therapy, thereby both preventing overtreatment/futile treatment and optimizing the management of TAO.

Extraocular muscle (EOM) is one of the main target tissue of TAO, its enlargement is the most characteristic pathological changes within the orbit (4, 7).Several magnetic resonance imaging (MRI)-based markers of EOMs, including the thickness of EOMs (8, 9), T2 signal intensity/ratio (T2-SI/SIR) of EOMs (10, 11) and volumes/cross-sectional areas of EOMs (12) have been found associated with the response. However, there are no uniformly valid markers to predict the curative efficacy of ivMP, and these conventional measures were either qualitative in nature or were based on morphologic changes.

T2 is a conventional MRI parameter describing tissue-specific time constant describing the decay of transverse magnetization of tissues (13). T2 relaxation time (T2RT) mapping can provide quantitative T2 values and demonstrates sensitivity in detecting subtle alterations of EOMs during the disease course (14) and in staging of TAO (15). However, the T2-mapping technique is currently not utilized to its full capacity. For example, previous studies mostly measured the average or highest T2RT on one or several slices of muscles (16), providing limited information and leading to sampling errors and subjective selection bias. Volumetric T2RT histogram analysis is based on T2 value distributions and measures the variations and frequencies of T2RT values within whole tissues (17). We hypothesized that T2 measures could reflect the subtle pathological changes of EOMs during disease progression, and could therefore be useful for predicting response to therapy

Accordingly, we aimed to predict the efficacy of ivMP therapy to TAO using baseline volumetric T2RT histogram features of EOMs. By analyzing the physical property of EOMs, we aimed to explore potential predictors of a good response to ivMP and to assist clinicians in selecting TAO patients who are truly benefited by ivMP.

## Materials and Methods

### Subject

The data analysis of this study was approved by the institutional review board of our hospital and the informed consent of this study was waived from the patient for the retrospective nature.

Adult patients who were diagnosed as active, moderate-to-severe TAO were included initially from the Endocrinology Department of hospital. Diagnosis and the activity stage of TAO was determined by an experienced ophthalmologist (Dr. Ban Luo) according to the guidelines of the European Group on Graves’ Orbitopathy (EUGOGO), 2016 (1). Active TAO was defined by: 1) clinical activity score (CAS) ≥3/7; or 2) CAS < 3/7, but with increased signal intensity of extraocular muscles on conventional T2 weighted images (T2WI). The CAS was recorded using a seven-point methods: spontaneous retrobulbar pain, pain on attempted eye movement, conjunctival hyperemia, eyelid redness, chemosis, swelling of the caruncle and swelling of the eyelid. The treatment regimen was as follows: 0.5 g methylprednisolone were administrated intravenously per week for 6 weeks, followed by 0.25 g per week for another 6 weeks. The total therapy course lasted for 12 weeks with the total dosage of methylprednisolone was 4.5 g. The therapeutic response was assessed within 2 weeks after the last treatment by the ophthalmologist Ban Luo.

The exclusion criteria were: (a) once underwent any other immunosuppressive therapy or radiotherapy before this ivMP treatment, (b) add radiotherapy or other treatment during the ivMP treatment course, (c) Non-compliance with weekly ivMP therapy according to the 2016 guidelines, (d) get lost for reassessment after therapy course, (e)compressive optic neuropathy or overt exposure keratopathy, and (f) poor image quality.

**Figure 1:**
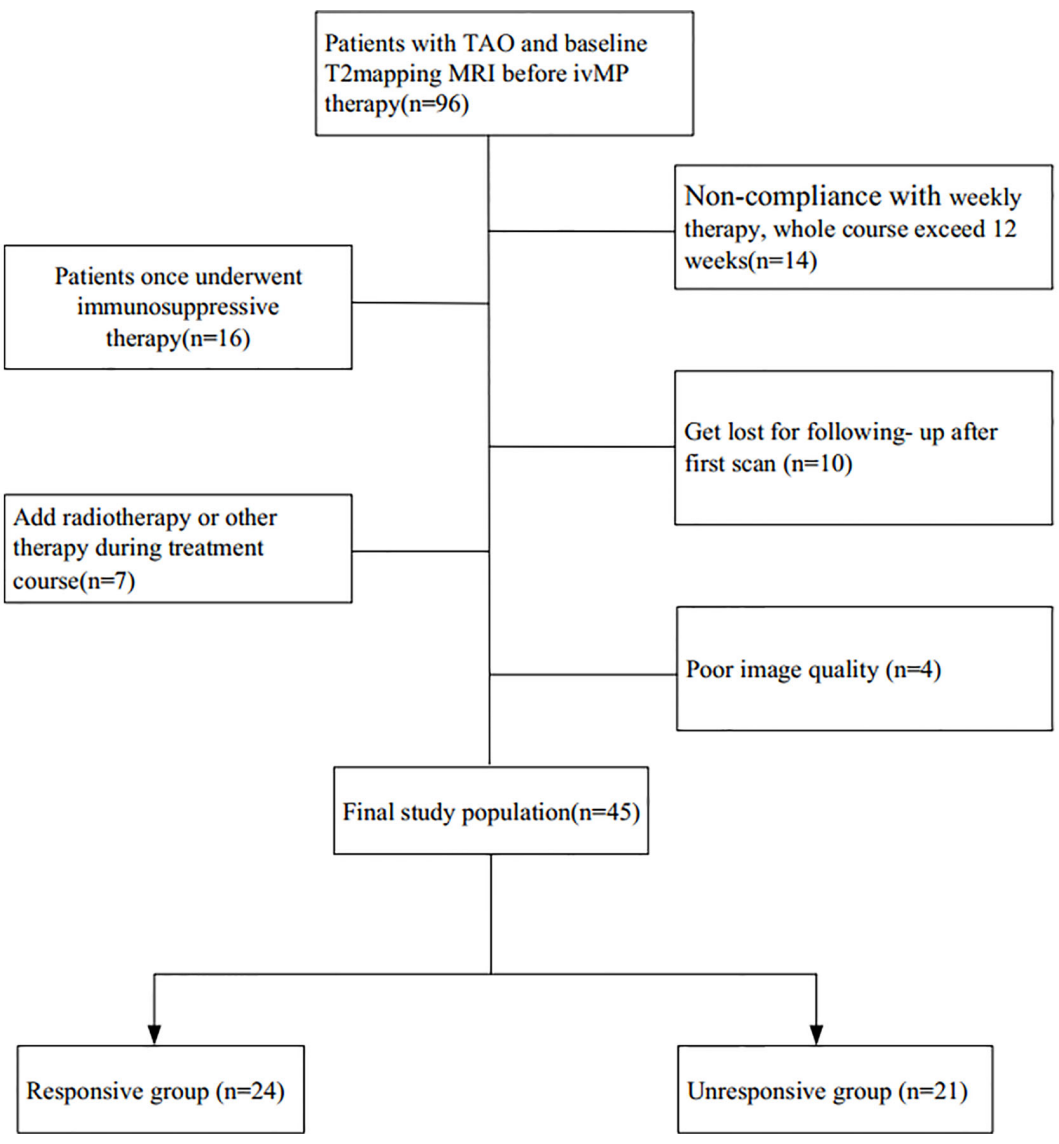
Flowchart of the patient enrollment process.

A flowchart of the patient enrollment is given in **Figure 1**. The participants’ clinical features, including age, gender, current thyroid function status, thyroid immune status, proptosis, intraocular pressure, CAS, and duration of TAO signs were investigated. Finally, 45 patients with bilateral TAO (25 women, 20 men) met the inclusion criteria.

### Orbital MRI

Orbital MRI was scanned within 1 week after the clinical examination and before the initial treatment. All MRI scans were performed on a 3T MRI scanner (Discovery 750, GE Healthcare, Milwaukee, WI) with a 32-channel head coil. The patients were placed in a supine position with their eyes closed during MRI scanning.

The MRI protocol included the following sequences: (a) axial T2WI image (repetition time [TR]/echo time[TE], 2800/68ms; slice thickness, 3 mm; intersection gap, 1 mm), (b) coronal T2-Ideal sequence (TR/TE, 2200/68 ms; slice thickness, 3 mm; intersection gap, 0.6 mm), and (c) coronal T2 mapping, which was performed using eight consecutive echoes (TE = 9.9–79.2 ms, ΔTE = 9.9 ms);TR, 2000 ms; section thickness, 3 mm; gap, 0.6 mm; field of view, 180x180 mm; matrix, 256x256; and bandwidth, 15.63 kHz/pix. Acquisition time for T2mapping was approximately 6 minutes 24 seconds.

### Therapeutic Efficacy Evaluation Based on the Clinical Index

The clinical evaluation of therapeutic response was performed within 2 weeks after completion of therapy (Dr Ban Luo, an ophthalmologist with 8 years’ experience in TAO) basing on three major, six minor criteria, according to previous studies (18). The major criteria were as follows: (1) improvement of at least 2 points in the CAS; (2) improvement in diplopia of at least 1 grade basing on the adapted NOSPECS classification 4, and (3) improvement of eye movement in any direction of 8° or more. The minor criteria were as follows: (1) reduction in exophthalmos by at least 2 mm; (2) remission of soft tissue involvement, according to the modified NOSPECS class 2 (19); (3) improvement in visual acuity by at least 1.0 Snellen unit; (4) decrease in CAS of 1 point; (5) reduction in lid width by at least 2 mm, and (6) improvement in diplopia (disappearance or degrade in degree). Patients who fulfilled at least one major or two minor criteria in any eye were classified into the “responsive group,” while those who did not meet this minimum threshold of responsiveness were classified into the “non-responsive group.”

### Image Analysis

Before quantitative measurements, conventional T2WI images were overviewed initially to confirm the most inflamed muscle by two experienced neuroradiologists (with approximately 4 and 7 years’ experience in head and neck radiology). The most inflamed EOM was defined on visual assessment by naked eye of the radiologist as: highest degree of increased brightness and swollen of EOM. Different opinions about the involvement evaluation were resolved by discussion.

All MR data from T2mapping were processed with an open source software (Fire Voxel, New York University, USA) for calculating the T2RT of five EOMs (superior, inferior, medial, lateral rectus, and superior oblique) within each orbit. One month after the determination of the most inflamed muscle, the two experienced neuroradiologists conducted the histogram analysis as follows:

1. The two neuroradiologists were blinded to the patients’ information, and independently drew region of interest (ROIs) slice by slice on the T2 map images (choosing a TE value that best depicts the EOM) along the edge of the EOMs to encompass the whole muscle. The ROIs were delineated carefully to avoid the partial volume effects from orbital fat and air in the paranasal sinuses.
2. After contouring all ROIs to cover the entire muscle, the VOIs (volume of interests) of the muscles were obtained. Repeating the same procedure, VOIs of the five EOMs within each orbit were generated. The superior rectus and levator palpebrae were sketched together as the superior muscle group due to the difficulty for separation.
3. Calculation of T2 relaxation times were conducted with mono-exponential T2mapping fitting model. For which, the relationship between signal intensity of T2maps and TE factors can be expressed by equation as following equation: S(TE) = *S*_0_ × *e* ^(-^ *^TE^* ^/^ *^T^* ^2)^. Where S represents the signal intensity (arbitrary unit), S0 is the initial signal intensity, TE is the echo time, T2 is T2 relaxation time (ms). Accordingly, the equations for skewness, kurtosis, entropy and inhomogeneity are as following:

$$skewness=\frac{\mu^{3}}{\sigma^{3}}=\frac{\frac{1}{N_{p}}\sum_{i=1}^{N_{p}}{\left(X\left(i\right)-\bar{X}\right)}^{3}}{{\left(\sqrt{\frac{1}{N_{p}}\sum_{i=1}^{N_{p}}{\left(X\left(i\right)-\bar{X}\right)}^{2}}\right)}^{3}}$$

$$kurtosis=\frac{\mu^{4}}{\sigma^{4}}=\frac{\frac{1}{N_{p}}\sum_{i=1}^{N_{p}}{\left(X\left(i\right)-\bar{X}\right)}^{4}}{{\left(\frac{1}{N_{p}}\sum_{i=1}^{N_{p}}{\left(X\left(i\right)-\bar{X}\right)}^{2}\right)}^{2}}$$

$$entropy=-\sum_{i=1}^{Ng}p\left(i\right)\log_{2}\left(p\left(i\right)+Є\right)$$

$$inhomogeneity=\sum_{i=1}^{Ng}p{\left(i\right)}^{2}$$

in these equations, the X be a set of N_p_ voxels included in the ROI, *p*(*i*) be the histogram with Ng discrete intensity levels, where Ng is the number of non-zero bins. For entropy, *є* is an arbitrarily small positive number (≈2.2×10^−16^).

4. Then, the histogram parameter of the T2RT were automatically calculated from each VOI, including (a) point-specific parameters: mean (representing average values within a specific ROI) and other cumulative T2 histograms: lower percentiles (5^th^, 10^th^, 25^th^, 50^th^), higher percentiles (75^th^, 90^th^, 95^th^), where the n^th^ percentile was the point at which n percent of voxel values were situated on the left area of the histogram (20, 21); and (2) histogram shape-related parameters, which indirectly reflected the heterogeneous distribution of T2: (a) standard deviation (SD, measuring degree of dispersion from the mean value); (b) skewness: reflecting asymmetry of the histogram distributions; (c) kurtosis: representing the peaked nature of the histogram; (d) entropy: describing irregularity or complexity of the distribution of a parameter in a specific region of interest (here, the entropy represents the first order features and it measures the average amount of information to encode the image values); and (e) inhomogeneity: quantifying intralesional heterogeneity.

### Statistical Analysis

Statistical analysis was performed using the SPSS statistical software (version 24, IBM Corp., Armonk, NY, USA). Data are expressed as means +/- SD or median (IQR) or number (proportion).were expressed as the mean ± standard deviation, with P <0.05 considered statistically significant.

The interclass correlation coefficient (ICC) was calculated to evaluate the measurement consistency between the neuroradiologists. The Kolmogorov– Smirnov test was conducted to test the normality of all continuous variables. Receiver operating characteristic (ROC) curves were used to determine Youden index-based cutoff values. Univariate analysis (χ^2^ test for categorical variables, independent sample *T*-test or Mann-Whitney *U* test for continuous variables) and binary logistic regression analysis were performed to identify significant predictors. The individualized nomogram model was developed based on the regression analysis results to predict response to therapy (R software: MathSoft, Cambridge, Massachusetts).

## Results

Totally, 45 patients with bilateral TAO (25 women, 20 men; mean age, 48.1 ± 11 years; range, 26–65 years) met the inclusion criteria. According to their treatment outcome patients were retrospectively divided into a “responsive group” (n = 24, 48 orbits) and an “unresponsive group” (n = 21, 42 orbits). None of the clinical features showed significant difference between the two groups on univariate analysis (**Table 1**).

**Table 1:** Univariate analysis of the clinical factors of TAO patients.

Variable	Responsive (N = 24)	Unresponsive (N = 21)	*P* value
Age	48 ± 10.63	48.19 ± 11.01	**0.950**
Gender (n, %)			**0.200**
Male	7(29.17%)	10(47.62%)	
Female	17(70.83%)	11(52.38%)	
Current thyroid status (n, %)			**0.245**
Euthyroid	11(45.83%)	8(38.1%)	
Hyperthyroid	5(20.83%)	9(42.86%)	
Hypothyroid	8(33.33%)	4(19.05%)	
Thyroid immune status (n, %)			**0.411**
Normal TRAb	4(16.67%)	5(23.8%)	
Abnormal TRAb	20(83.33%)	16(76.19%)	
CAS (median) (Q25, Q75)	4(2,3.75)	4(2,4)	**0.603**
Duration of TAO(months) (median) (Q25, Q75)	5.5 (2,10.75)	6(4,9)	**0.511**
Proptosis (mm)	18.5 ± 2.9	19.6 ± 2.95	**0.092**
Intraocular pressure (mm Hg)	19.98 ± 3.95	18.41 ± 3.15	**0.065**

Values are expressed as means +/- SD or median (IQR) or number (proportion).

TRAb, Thyrotropin receptor antibody.

CAS, Clinical activity scores.

The current thyroid status was judged according to the serologic test within 2 weeks before MRI examination.

The duration of eye signs was the time interval between the onset of abnormal symptoms based on the patient’s complaint and the time to consultation. The values of the result were displayed in bolded text.

### Histogram Parameters Analysis

The degree of inter-observer agreement was excellent (ICC > 0.822) for all histogram parameters (**Supplementary Table 1**). Therefore, the measurements from one of the radiologist were chosen to perform further analysis.

Based on the cutoff values from ROC analysis (**Table 2**), the continuous variables were split into two mutually exclusive categories.

**Table 2:** The ROC of the T2RT histogram parameters.

Parameters	AUC value	*p*	Youden	Cutoff value	Sensitivity (%)	Specificity (%)
T2RT_5%_	0.561 (0.509, 0.611)	0.041	0.113	≤56.25	56.77	54.45
T2RT_10%_	0.549 (0.498, 0.600)	0.092	0.102	≤58.1	48.96	61.26
T2RT_25%_	0.518 (0.467, 0.569)	0.553	0.059	≤67.25	68.75	37.17
T2RT_50%_	0.525(0.469, 0.571)	0.503	0.086	>69.5	66.67	41.88
T2RT_mean_	0.508(0.457, 0.559)	0.790	0.070	>70.8	68.75	38.22
T2RT_75%_	0.556 (0.505, 0.620)	0.060	0.138	>76.25	69.79	43.98
T2RT_90%_	0.582(0.525, 0.639)	0.005	0.169	>81.9	79.17	37.7
T2RT_95%_	0.660(0.606,0.715)	<0.001	0.403	>88.1	86.98	43.46
Skewness	0.633(0.583,0.682)	<0.001	0.301	>0.31	75	54.97
Kurtosis	0.521 (0.469, 0.572)	0.492	0.069	≤0.598	74.48	32.46
Entropy	0.745 (0.695, 0.794)	<0.001	0.404	>3.41	76.56	63.87
Inhomogeneity	0.681 (0.632,0.727)	<0.001	0.305	>0.136	80.21	50.26

AUC, area under curve.

T2RT, T2 relaxation time.

The unit of T2RT is ms.

### Univariate Analysis

**Figure 2:**
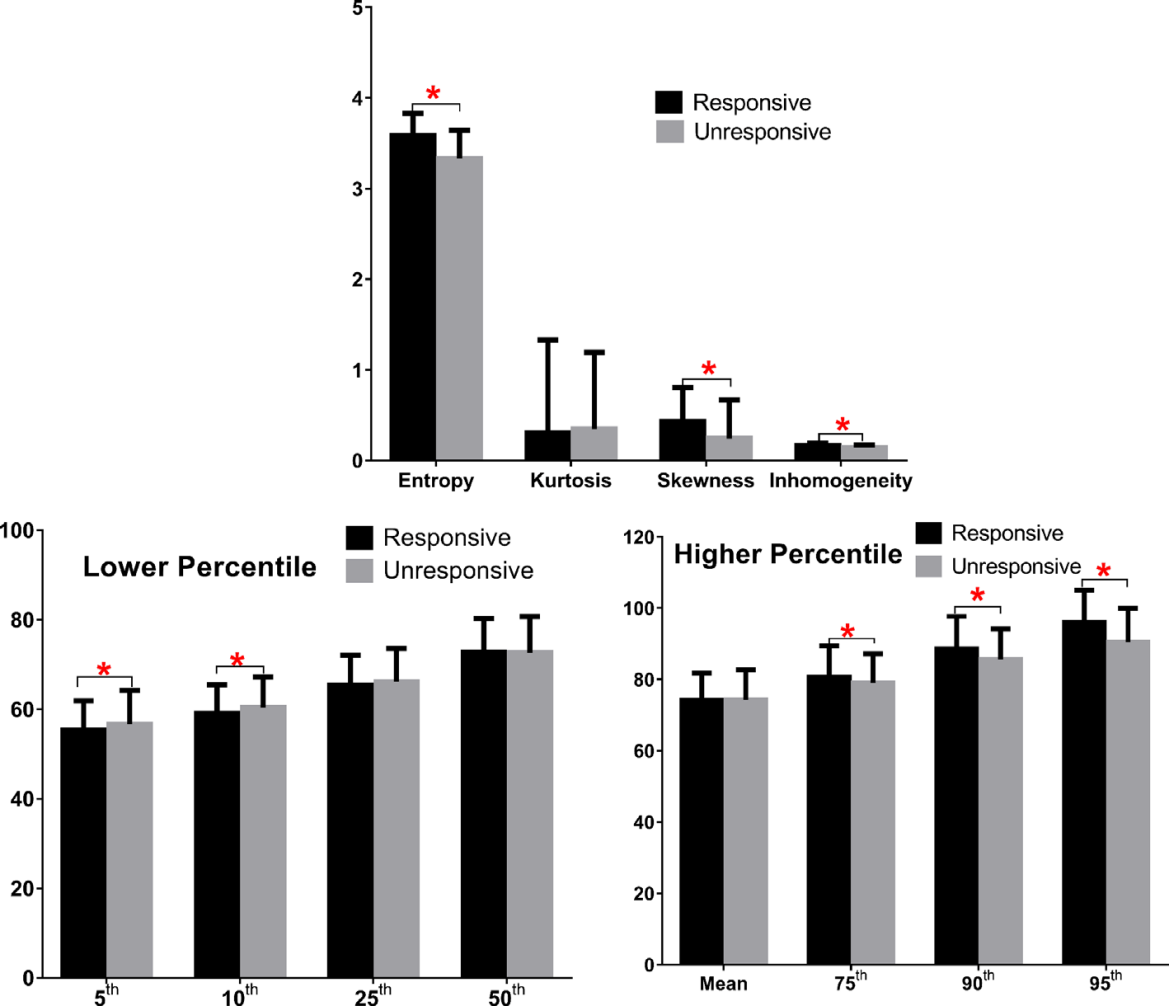
Comparison of baseline histogram-based texture metrics of EOMs for the two groups of patients with TAO. The * stand for the p value of the comparison was less than 0.05, or, had significant statistical difference

As shown in **Figure 2** and **Table 3**, eight predictors including lower (5^th^ and 10^th^) and higher (75^th^, 90^th^, and 95^th^) percentiles of T2RT, skewness, entropy, and inhomogeneity displayed a significant association with good response to ivMP therapy.

**Table 3:** Univariate analysis of the histogram parameters on the response rate of ivMP.

Variable	Responsive (N = 48)	Unresponsive (N = 42)	P value
T2RT_5%_, n (%)			0.031
>56.25	21(47.7%)	23(52.3%)	
≤56.25	27(58.7%)	19(41.3%)	
T2RT_10%_, n (%)			0.040
>58.1	25(49.1%)	26(50.9%)	
≤58.1	23(58.9%)	16(41.1%)	
T2RT_25%_, n (%)			0.223
>67.25	15(48.4%)	16(51.6%)	
≤67.25	33(55.9%)	26(44.1%)	
T2RT_50%_, n (%)			
>69.5	32(57.1%)	24(42.9%)	0.081
≤69.5	16(47.1%)	18(52.9%)	
Mean			
>70.8	33(55.9%)	26(44.1%)	0.149
≤70.8	15(48.4%)	16(51.6%)	
T2RT_75%_, n (%)			
>76.25	34(58.6%)	24(41.4%)	0.005
≤76.25	14(43.8%)	18(56.2%)	
T2RT_90%_, n (%)			<0.001
>81.9	38(63.3%)	22(36.7%)	
≤81.9	10(33.3%)	20(66.7%)	
T2RT_95%_, n (%)			<0.001
>88.1	42(63.6%)	24(36.4%)	
≤88.1	6(25%)	18(75%)	
Skewness, n (%)			<0.001
>0.31	36(65.5%)	19(34.5%)	
≤0.31	12(34.3%)	23(65.7%)	
Kurtosis, n (%)			0.168
>0.598	12(46.2%)	14(53.8%)	
≤0.598	36(56.3%)	28(43.7%)	
Entropy, n (%)			<0.001
>3.41	37(71.2%)	15(28.8%)	
≤3.41	11(28.9%)	27(71.1%)	
Inhomogeneity, n(%)			<0.001
>0.136	38(61.3%)	24(38.7%)	
≤0.136	10(35.7%)	18(64.3%)	

T2RT, T2 relaxation time. The unit of T2RT is ms.

### Binary Logistic Regression Analysis

**Figure 3:**
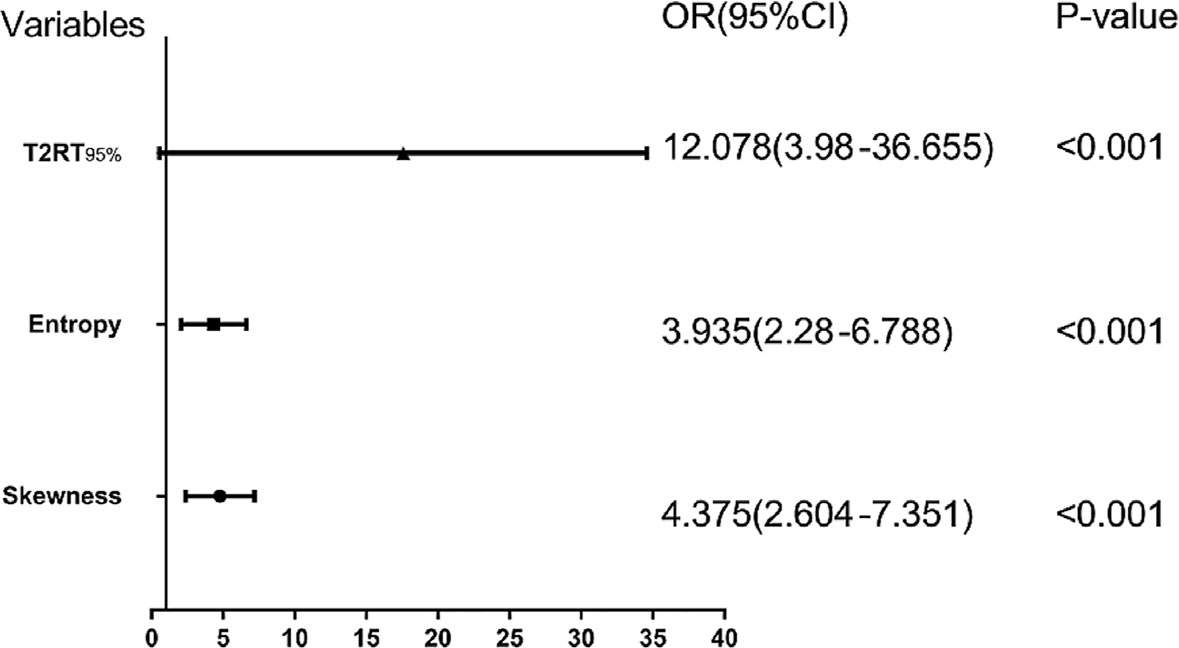
Forest plot of each predictor. The left column lists the names of the predictors. The odds ratio for each of these studies is represented by a square, and confidence intervals are represented by horizontal lines. CI, confidence interval; OR, odds ratio.

Eight predictors from univariate analysis were entered into this step, but only 3 predictors, including 95^th^ percentile >88.1, skewness >0.31, and entropy >3.41 were significantly related to a favorable response, with odds ratios (ORs) of 12.08, 4.38, and 3.94, respectively (**Table 4**, p < 0.05). In addition, the result of the multivariate analysis was visualized as a forest plot (**Figure 3**).

**Table 4:** Binary logistic regression model for prediction of the therapy response to ivMP.

Variables	β	SE	OR	95% CI	P*
T2RT_5%_≤56.25	0.165	0.489	1.18	0.453–3.074	0.737
T2RT_10%_≤58.1	0.919	0.506	2.51	0.93–6.758	0.065
T2RT_75%_>76.25	0.172	0.469	1.19	0.474–2.976	0.709
T2RT_90%_>81.9	-1.144	0.632	0.32	0.092–1.099	0.068
T2RT_95%_>88.1	2.491	0.566	12.08	3.98–36.655	<0.001
Skewness>0.31	1.476	0.265	4.38	2.604–7.351	<0.001
Entropy>3.41	1.37	0.278	3.94	2.28–6.788	<0.001
Inhomogeneity>0.136	0.349	0.305	1.42	0.78–2.576	0.249
Consistent	-3.518	0.467	0.03	–	<0.001

β, coefficient from binary logistic regression model.

OR, odds ratio; SE, standard error; CI, confidence interval.

T2RT, T2 relaxation time. The unit of T2RT is ms.

### Development of an Individualized Prediction Nomogram

**Figure 4:**
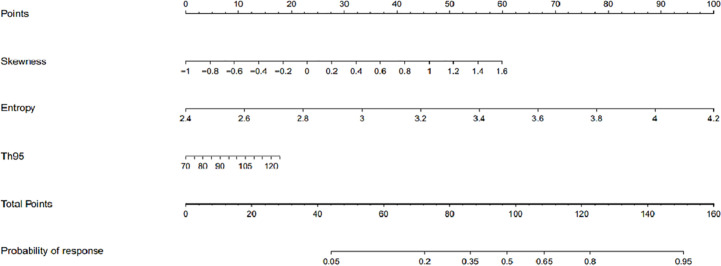
The developed nomogram for prediction of efficacy of intravenous methylprednisolone pulse (ivMP) therapy for thyroid-associated ophthalmopathy (TAO), incorporating the 95th percentile T2 relaxation time (T2RT), skewness, and entropy. In this nomogram, entropy is the greatest predictor of therapeutic response (100 points), followed by skewness (60 points), and 95^th^ percentile T2RT (18 points).

**Figure 5:**
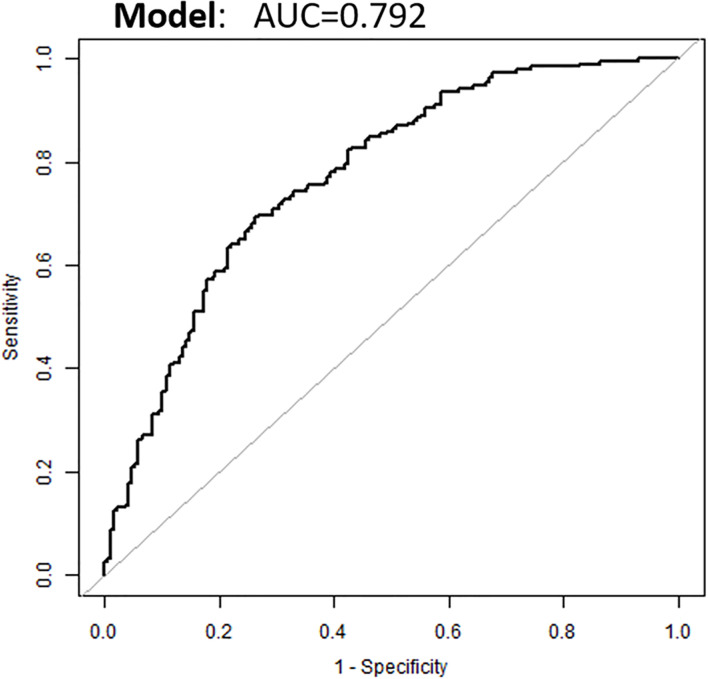
Receiver operating characteristic curve of the nomogram.

Based on the above binary logistic regression analysis, the three independents predictive factors were used to construct a nomogram predicting the cumulative probability of ivMP therapy response (**Figure 4**). The model showed good accuracy, with a C-index of 0.792 (95% CI, 0.720–0.839) (**Figure 5**). The values of the three predictors for an individual patient are located on each variable axis of the nomogram, and then a line is drawn upwards to determine the points received for each variable value for usage. The corresponding therapy response rate was listed in **Supplementary Table 2**).

**Figure 6:**
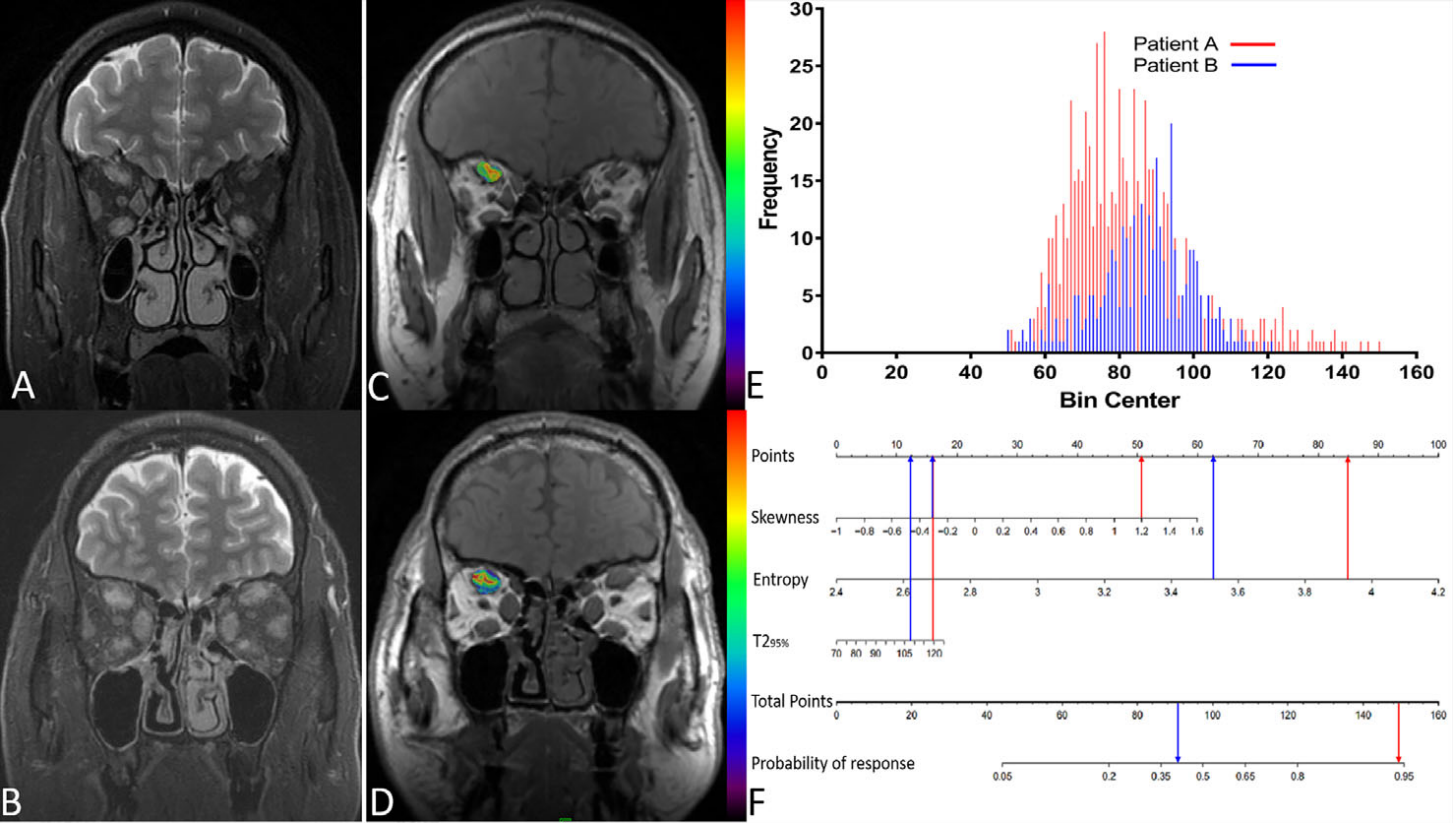
**(A)** A 51-year-old male patient**(A)** with thyroid-associated ophthalmopathy (TAO); **(B)** A 48-year old male patient **(B)** with TAO. Both of them have similar clinical features and accepted identical ivMP treatment. Patient A showed a good response but patient B showed a poor response. **(C**, **D)** The overlap of T2 histograms of patient A and B, respectively. **(E)** Composite histogram distribution of the T2 values of patient A and B; the red bar represents the responders, while the blue bar represents the non-responders. **(F)** Individuated nomogram for therapy response prediction. The red arrows presented the corresponding points of each predictor for responders and the total points was about 101.5 with the response rate is close to 95%. The blue arrows presented the points of each predictor for poor responders and the total points was about 90.75, the approximate response rate is lower than 40%.

**Figure 6** displays two TAO patients with favorable and poor therapeutic responses, and the histogram analysis and the corresponding points on the response prediction nomogram.

## Discussion

In this study we employed a voxel-wise analysis of T2RT values of EOMs to predict the response to ivMP therapy for TAO. Our results demonstrated that: (1) patients with a favorable response displayed a more heterogeneous T2RT distribution; (2) baseline 95^th^ percentile T2RT, skewness, and entropy were effective predictors of therapeutic response.

Efforts have been made to identify the predictive biomarkers of response to treatment, such as glucocorticoid receptor gene polymorphisms (22), serum levels of antibody (23), and miR-224-5p (24). However, none of them reflect the biology information of target tissue. Based on previously reported differences in clinical features between responders and non-responders (Hu et al., 2020) and the current results, we found that baseline clinical features do not always effectively differentiate responders from non-responders, especially in TAO patients who have similar disease states and receive identical standard therapeutic regimens.

In this study, we predicted therapeutic responses based on underlying pathologic properties of the target tissue by analyzing the T2RT histogram features of EOMs. In TAO, the heterogeneous cellular and molecular expression, including fibroblast phenotypes, cytokine profiles, and disparate T cell subsets (25, 26), may produce asynchronous and non-uniform pathological changes in each EOM within the same orbit, thus the disease distribution within each EOM may be uneven. The varying deposition of macromolecules (e.g., collagen), interstitial edema, and fibrous scar tissue within the involved muscles can result in greater distribution of T2RTs (27). Histogram features provide novel quantifications of the intrinsic distributions of different tissues; for example, the percentiles can demonstrate a greater range of T2RT values to reveal complex tissues. In fact, an early study (28) noticed that the nonuniformity on T2 images may relate to treatment response for TAO, and they ascribed the nonuniformity to the coexistence of different pathology. However, the report was based on T2 signal intensity from visual observation, was subjective and unable to quantify the tissue properties.

The results of this study showed that patients with favorable response displayed larger values in higher T2RT percentiles (75^th^, 90^th^, and 95^th^) than those of unresponsive group. The T2RT is related to the amount of free (extracellular) water (as seen in most edematous tissues) and its interaction with the neighboring environment (29). In immunologically active stage, a large amount of glycosaminoglycans are produced, causing water accumulation and obvious edema of EOMs (26) and resulting in markedly prolonged T2 relaxation times. In histograms, the T2RT values were arranged from small to large. Higher percentiles represent those voxels with higher T2 value, and may indicate more tissue components and more complex environment. Thus, we speculate tissues with greater T2RT have more glycosaminoglycans and are therefore more amenable to response than tissues containing less soluble fibrotic compounds. Therefore, patients with larger higher percentiles would obtain dramatic amelioration in response to ivMP (30), especially the 95^th^ percentile T2RT. This were also in accordance with the therapeutic mechanism of action of steroids, including anti-inflammatory, immunosuppressive and glycosaminoglycan reducing mechanisms (31). The findings are also in agreement with Ohnishi T et al. (16, 32) who found that higher T2RTs were correlated with a superior therapeutic response, but the T2RTs in that report were generated from mean value, which is not utilized in its full capacity to provide the distribution of different T2RT in a given tissue.

Histogram shape-related parameters refer to the indexes reflecting the shape and general features of the histogram distribution of a given volume of interest (VOI). Skewness reflects the asymmetry (33); it is positive if most of the data lie to the right of the mean, and a higher value indicates more voxels with high T2RT value within the VOI. The inhomogeneity quantifies the heterogeneity of a specific tissue. The entropy refers to irregularity of T2 value distribution, higher entropy reflects greater complexity (34). In TAO, the biopsy of EOMs demonstrated the coexistence of histopathological changes at different stages, including interstitial edema, mucopolysaccharide deposition and fibrous tissue (27, 35). Higher entropy indicates the diversity tissue component, further suggesting that the pathology in EOMs is in dynamic progression rather than in a stable fibrotic phase.

ROC analysis demonstrated that entropy had the highest AUC; in the personalized nomogram, its subtle change can bring great changes for response probability and maybe the most sensitive predictor of therapeutic response. Whereas, the 95^th^ percentile T2RT, bringing slightly alteration of prediction probability with a large scale increase of values, indicate it weigh little for therapy response prediction. This mean that the complexity of biological components of EOMs weigh more crucial than the degree of edema in response prediction. The results are also in agreement with Yokoyama N’s report (28), in which the uniformity of MRI-T2 pattern (low SI area in T2) was actually identical to the complex tissue component and was viewed as a predictor for response. Although they recognized the uniformity of the T2 pattern from distinct pathology changes, but they could not quantify it and provide objective and convincing evidence. Varying degrees of immune infiltrates between individual EOMs lead to distinct biochemical components of EOMs and heterogeneous T2RT distributions; these differences explain the wide variability in therapeutic responses in patients with TAO. Thus, a nomogram model incorporating multi-parametric T2RT histogram possesses optimal prediction efficacy, suggesting that the extent and heterogeneity of the pathology area jointly determined the therapeutic response.

Notably, if this therapy response prediction modal and individualized nomogram could be employed clinically, the therapy effect of every TAO patient can be evaluated before initial treatment. Consequently, those patients who have a totally lower response probability to ivMP before initial therapy can be switched to other anti-inflammatory treatment or be added with radiotherapy, or other novel treatment including insulin-like growth factor I receptor (6, 36, 37), interleukin-1-receptor antagonists or soluble interleukin-1 receptors (38), or several other regimens can be combined to achieve the optimal intervention. Thus, avoiding the waste of other additional therapies in responders and unnecessary potentially toxic ivMP in non-responders.

Our study does have some limitations. First, the retrospective nature, a single-center study and the small sample size may reduce the statistical power of our study. Consequently, failed to perform truly artificial intelligent or machine learning, together with no cross-validation analysis to assess the generalizability of the results may discount the conviction. Therefore, a future prospective, multicenter study with a larger sample size is needed to obtain conclusive evidence of our findings. Second, other therapy regimens were not included for comparison; we think that comparison group would bring more value to this study, and in fact, this research is ongoing. Third, the absence of a quantification of pathologic or immunohistochemical changes as reference standard, and we could not correlate the histogram T2RT parameters with pathological findings, particularly the quantification of cellular components. Fourth, the slice gap and thickness render the T2-mapping technique not fully realize whole-volume analysis. Lastly, combination of T2 and histogram analysis is still failed to differentiate active inflammation and congestion.

## Conclusion

This study shows that volumetric T2RT histogram analysis could be used as non-invasive objective surrogate biomarkers for future therapeutic trials. It may be of great value in selecting TAO patients who are good candidates for ivMP therapy. Such a baseline nomogram prediction model is crucial for individualized treatment, by providing an opportunity to modify the therapy regimen before initial therapy, ultimately empowering the clinicians to provide better patient care.

## Data Availability Statement

The raw data supporting the conclusions of this article will be made available by the authors, without undue reservation.

## Ethics Statement

The studies involving human participants were reviewed and approved by Ethics Committee of Tongji Medical College. The patients/participants provided their written informed consent to participate in this study.

## Author Contributions

JZ and BL were involved in the conception and design of the study. PL, BL, LC, Q-XW, and GY were involved in the data collection. JZ and PL were involved in the analysis and interpretation of the data. PL wrote the first draft of the manuscript. JZ, BL, and G-hJ revised the article critically for important intellectual content. All authors contributed to the article and approved the submitted version.

## Funding

This work was supported by grants from the National Natural Science Foundation of China (No.81771793), Young science foundation of Guangdong Second Provincial General Hospital (2019-QNJJ-01), and the Natural Science Foundation of Hubei Province, China (No. 2017CKB900).

## Conflict of Interest

The authors declare that the research was conducted in the absence of any commercial or financial relationships that could be construed as a potential conflict of interest.
