# Supplementary material for: Baseline Volumetric T2 Relaxation Time Histogram Analysis: Can It Be Used to Predict the Response to Intravenous Methylprednisolone Therapy in Patients With Thyroid-Associated Ophthalmopathy?
Source: Front Endocrinol (Lausanne). 2021 Feb 25;12:614536. doi: 10.3389/fendo.2021.614536 (PMC7947366; doi:10.3389/fendo.2021.614536)
Supplement: Supplementary file 1 [file Table_1.docx]

Supplementary Material

# Supplementary Tables

**Table 1:** The Interobserver agreement between two radiologists of different T2RT histogram analysis parameters.

| Parameters | ICC | 95%CI |
| --- | --- | --- |
| T2RT_5%_ | 0.889 | 0.836-0.926 |
| T2RT_10%_ | 0.914 | 0.872-0.942 |
| T2RT_25%_ | 0.930 | 0.907-0.948 |
| T2RT_50%_ | 0.913 | 0.885-0.935 |
| T2RT _mean_ | 0.935 | 0.913-0.951 |
| T2RT_75%_ | 0.894 | 0.831-0.933 |
| T2RT_90%_ | 0.917 | 0.890-0.937 |
| T2RT_95%_ | 0.822 | 0.718-0.888 |
| Skewness | 0.901 | 0.853-0.933 |
| Kurtosis | 0.908 | 0.863-0.938 |
| Entropy | 0.960 | 0.941-0.973 |
| Inhomogeneity | 0.906 | 0.843-0.943 |

**Note:**

ICC, intraclass correlation coefficient. CI, confidence intervals.

T2RT, T2 relaxion time.

**Table 2:** Prediction of ivMP therapy response to each score

| Response rate |  | Scores |
| --- | --- | --- |
| 0.05 |  | 44 |
| 0.2 |  | 72 |
| 0.35 |  | 86 |
| 0.5 |  | 97 |
| 0.65 |  | 109 |
| 0.8 |  | 123 |
| 0.95 |  | 151 |

**
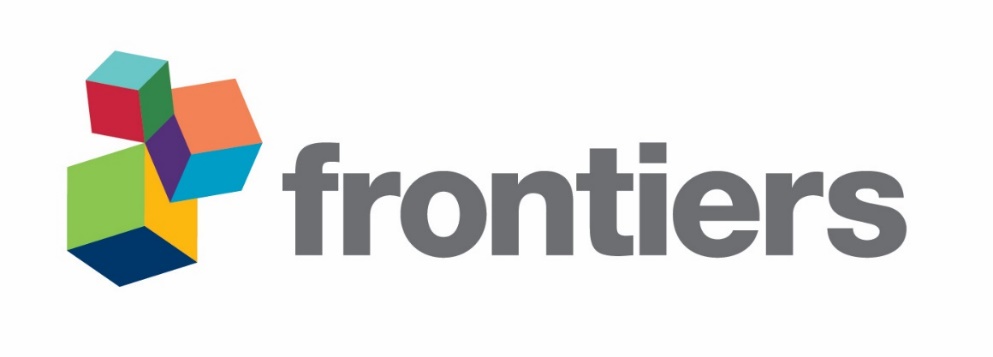
**
